# GinDB-AI: An integrated ginsenoside database and AI-driven platform for multidimensional information and biological activity prediction

**DOI:** 10.1016/j.jgr.2026.100986

**Published:** 2026-02-04

**Authors:** Nguyen Doan Hieu Nguyen, Vinoth Kumar Sangaraju, Duong Thanh Tran, Nhat Truong Pham, Jae Youl Cho, Balachandran Manavalan

**Affiliations:** a Department of Integrative Biotechnology, College of Biotechnology and Bioengineering, Sungkyunkwan University, Suwon, 16419, Gyeonggi-do, Republic of Korea; b Department of Biocosmetics, Sungkyunkwan University, Suwon, 16419, Republic of Korea

**Keywords:** Ginseng database, Deep learning, Physicochemical properties, Biological functions

## Abstract

GinDB is a comprehensive database containing 753 standardized ginsenosides with curated and corrected data from 1963 to 2024. Integrated with GinDB-AI, a deep learning module for predicting bioactivity and physicochemical properties, the platform enables rapid structure-activity analysis and visualization. The freely accessible resource accelerates ginsenoside-based drug discovery and natural product research.

## Graphical abstract

**Figure:**
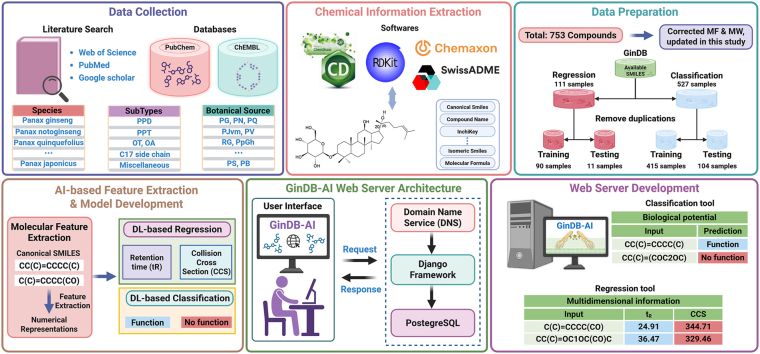

## 1 Introduction

Ginseng is one of the most extensively studied medicinal plants, with centuries of use in traditional medicine, especially in East Asian countries [1], and increasingly recognized in modern pharmacology. Despite decades of study, knowledge about ginsenosides remains fragmented and difficult to access. General small-molecule databases (DrugBank,^2^ ChEBI,^3^ and PubChem^4^) contain only a small subset of ginsenosides and rarely provide compound-specific analytical or functional annotations. To meet the pressing need, several domain-specific databases have been developed. [2], compiled 289 triterpenoid saponins from 11 *Panax* species between 1963 and 2012, and categorized them into sapogenin-based subtypes, [3] subsequently updated these data with biological function, metabolism, and methodological advances, PanaxGDB [4] integrated multi-omics data and provided analytical tools, containing 587 compounds from 12 *Panax* species, and more recently, GinMIL [5] has offered an integrated database of ginsenosides, including additional information on spectrometric and chromatographic parameters, and integrating machine learning (ML)-based approaches for data imputation to address missing values. However, there remain significant concerns regarding inaccuracies in the reported substance information, such as discrepancies in the molecular formula of ginsenosides, as well as deficiencies in the visualization of the molecular structure of these compounds.

In this study, we introduce the GinDB-AI, a unified and AI-enabled platform integrating a rigorously curated ginsenoside database with predictive modelling for physicochemical properties and biological functions. We systematically curated the literature from 1963 to 2024 and compiled data with stereochemically explicit identifiers and structures, generating missing alongside reputable databases (PubChem and ChEMBL^5^). Using ChemDraw^6^ and RDKit,^7^ we generated missing molecular strings (canonical SMILES [6]) and corresponding structural representations, thereby enriching the database with essential information about ginsenosides. Based on this curated database, we developed deep learning (DL) models comprising two regression models to predict t_R_ and CCS values, and a classification model for biological function prediction. Through extensive cross-validation and independent testing, we identified optimal models for each task, forming useful tools for the database. This platform is accessible at https://balalab-skku.org/GinDB-AI/, offering a user-friendly web interface for querying, browsing, and analysing multidimensional molecular information and biological functions of ginsenosides. We anticipate that GinDB-AI will accelerate discovery and mechanistic understanding of ginsenosides and support applications spanning food chemistry, natural products research, pharmacology, and clinical applications related to ginseng.

## 2 Materials and methods

### 2.1 Database construction

**Fig. 1:**
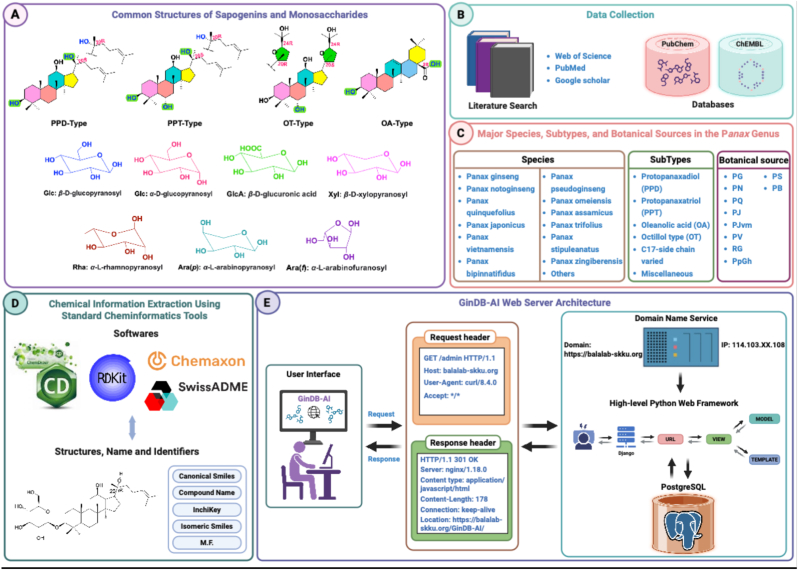
Construction of GinDB. (A) Common structures of sapogenins and monosaccharides. (B) Literature and databases used for data collection. (C) Major species, subtypes, and botanical sources in the Panax genus. (D) Chemical information extraction using standard cheminformatics tools. (E) Web server architecture of GinDB.

We conducted a comprehensive literature and database search spanning 1963 to 2024 using PubMed, Google Scholar, and the chemical repositories PubChem and ChEMBL. This meticulous process led to the identification of 753 ginsenoside compounds from 12 *Panax* species and other miscellaneous sources. To enhance data organization, ginseng species were categorized based on their subtypes and botanical resources (Fig. 1). For each compound, we generated its 2D chemical structures using ChemDraw (version 23.1.1) and its corresponding standardized canonical SMILES string using RDKit (version 2023.9.6). The structural consistency was visually verified using SwissADME [7] (Fig. 1). To ensure data accuracy, we carefully verified the molecular formulas, molecular weights, chemical structures, and related metadata of all collected compounds. The rigorous validation uncovered inconsistencies or errors in 48 compounds found in existing databases and literature, which were corrected in GinDB (Table S1).

To standardize input representations, we generated non-isomeric canonical SMILES, which would be helpful for downstream tasks, especially while developing the models. Our choice is consistent with previous studies, which demonstrated that stereochemical tokens in SMILES can be order-dependent and do not improve prediction performance in limited or heterogeneous datasets [8].

GinDB is deployed on a modern web infrastructure built for accessibility and performance. The backend data was managed by PostgreSQL (version 12.20) running on Nginx web server (version 1.18.0). The front end was implemented using HTML5, Bootstrap (version 5.0.1), CSS (version 3), and JavaScript to deliver responsive interfaces compatible with mobile, tablet, and desktop devices. The application layer was built in Django (version 4.0.4).

### 2.2 Development of prediction tools

Of 753 curated ginsenosides in GinDB, 111 samples have experimentally reported multidimensional attributes, and 527 ginsenosides have available bioactivity data (Fig. S1). The substances are in SMILES [6] format, which was incompatible with training the DL models. To convert the data into numerical representations, we implemented some preprocessing steps, including removing redundant samples and retaining only one entry per unique SMILES string, splitting the datasets into training and testing sets, and scaling the continuous targets for a better target distribution (Text S1 and S2). We then employed one molecular descriptor, 11 molecular fingerprints, and seven pretrained natural language models (Text S3) to extract numerical features from the input molecular string representations (SMILES or SELFIES [9], which is encoded from SMILES). Apart from the SMILES, the regression dataset was complemented with the adduct information, specifically [M−H]^-^ (deprotonated) and [M−H + HCOOH]^-^ (formate adduct). To account for ionization states, we include this information in the training process of the DL-based tools with the aim of enhancing the models’ performance (Text S4).

We designed the DL models for the regression and classification tasks (Text S4 and S5), as described in Fig. S1C and S1D, respectively, and trained them using 10-fold cross-validation combined with a grid search mechanism to identify the optimal configuration yielding the highest performance (Text S6). To evaluate the performance of the models, we employed the popular metrics used in related research for regression and classification tasks (Text S7).

## 3 Results and discussion

### 3.1 Database structure and statistics

**Fig. 2:**
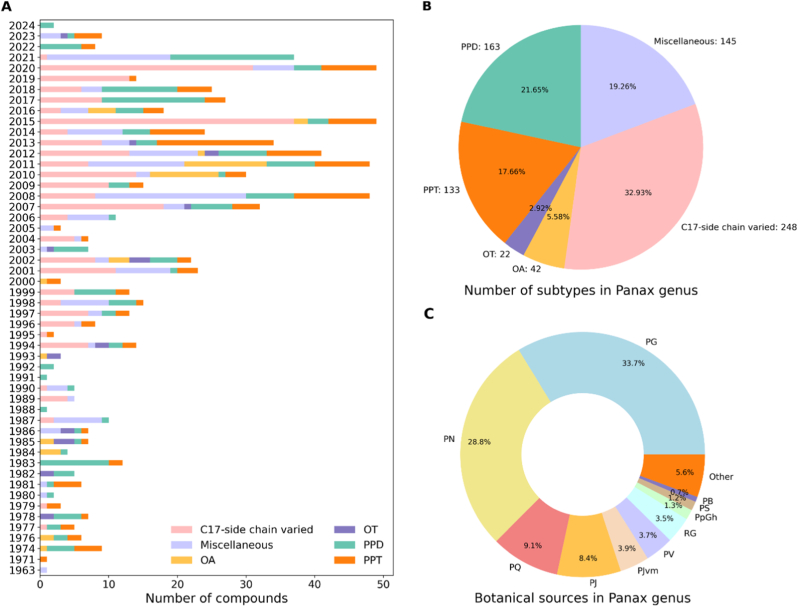
GinDB statistics. (A) A comprehensive timeline from 1963 to 2024 detailing saponin and non-saponin compounds discovery, including their subtypes within the Panax genus. (B) The pie chart depicts the distribution of subtypes within the Panax genus. (C) The diverse botanical sources of Panax saponins, showcasing the genus's rich diversity.

GinDB contains a total of 753 ginsenoside compounds, derived from various *Panax* species. Each entry was meticulously compiled from peer-reviewed literature and well-established databases. The ginsenosides were systematically classified into six structural subtypes based on the core skeletons. The largest group, representing 32.93% (248) compounds belong to the C17-side chain varied group, 21.65% (163) compounds are classified as protopanaxadiol (PPD), 17.66% (133) compounds as protopanaxatriol (PPT), 5.58% (42) compounds as oleanolic acid (OA), 22 (2.92%) compounds as octillol type (OT), and 145 (19.26%) compounds fall under the miscellaneous category (Fig. 2B). The botanical sources of these ginsenosides were also analyzed, revealing that *Panax* ginseng is the primary contributor, accounting for 33.7% of the compounds. It was followed by *Panax* notoginseng with 28.8%, *Panax* quinquefolius at 9.1%, and *Panax* japonicus at 8.4% (Fig. 2C). The distribution highlights the key species that serve as a source for the majority of known ginsenosides. An analysis of historical discovery trends (Fig. 2A) shows a consistent and increasing rate of new ginsenoside identification over recent decades, with a notable surge in discoveries between 2007 and 2021. GinDB-AI currently contains 753 ginsenosides, representing an approximately 28% increase in the number of compounds compared to PanaxGDB (587 compounds, reported as of 2022) and a 30% increase compared to GinMIL (579 compounds, reported as of 2024). These additions substantially expand compound coverage and support broader downstream analysis and prediction.

Despite the comprehensive nature of GinDB, a significant challenge in ginsenoside research is the scarcity of annotated multidimensional and functional data. Our curation revealed that only ∼15% of the ginsenosides have experimentally determined multidimensional information (CCS, t_R_), and 28.5% of compounds lack reported biological activity. This data sparsity limits the utility of traditional database approaches. To overcome this critical gap, we developed a suite of DL models integrated directly into the GinDB platform.

### 3.2 Deep learning models’ performances

#### 3.2.1 Predicting multidimensional information

Following the evaluation process, we determined that the DL model utilizing Mol2vec features, enhanced by adduct information, demonstrated remarkable performance with a training R^2^ of 0.9385 and a testing R^2^ of 0.9143 (Text S8, Table S5, and Table S6). This configuration outperformed all others, establishing it as the optimal choice for predicting the CCS.

Conversely, the Estate descriptor proved to be essential for t_R_ prediction, yielding a training R^2^ of 0.7462 and a testing R^2^ of 0.8774 (Text S8, Table S7, and Table S8). The inclusion of adduct information was found to diminish prediction accuracy, suggesting its irrelevance to t_R_. Consequently, excluding adducts led to more precise predictions in the final DL model for predicting t_R_.

#### 3.2.2 Biological activity classification

The model using Mol2Vec features achieved the highest metrics with an MCC of 0.5276, outperforming other configurations (Text S9 and Table S9). While some models struggled with generalization, the Mol2Vec model maintained strong transferability and notable performance improvements. Overall, this combination showcases high accuracy and robust generalization, making it ideal for biological classification tasks.

### 3.3 Web server functions

To ensure widespread accessibility and practical usability of GinDB-AI, we have deployed a user-friendly web server at https://balalab-skku.org/GinDB-AI/. The platform integrates three distinct prediction models: two for multidimensional property prediction and another for biological activity classification of ginsenosides. This modular architecture enables users to effectively interrogate diverse aspects of ginsenoside functionality from a single interface. To promote reproducibility and facilitate benchmarking, we provide comprehensive datasets used in the development and validation of GinDB-AI through our downloads page at https://balalab-skku.org/GinDB-AI/downloads/. This includes the full curated data table comprising structural, physicochemical, and functional annotations of all ginsenosides analyzed in this study.

A comprehensive user guide is available at https://balalab-skku.org/GinDB-AI/userguide/, providing step-by-step instructions for data formatting, submission, result interpretation, and retrieving submitted jobs. Detailed information for each compound can be accessed at https://balalab-skku.org/GinDB-AI/Compounds/, allowing users to explore chemical structures and properties. It is important for users to format their input molecules in SMILES format prior to server submission, and we provide examples to guide users through this process. Once a submission is made, the results are displayed in table format, and users can download these results as CSV files for further analysis. By integrating predictive modeling with intuitive design and comprehensive documentation, GinDB-AI offers a robust and accessible platform for researchers engaged in the computational exploration and functional annotation of ginsenosides.

## 4 Conclusions

GinDB-AI is a comprehensive and intelligent platform that addresses the longstanding limitations in ginsenoside research by combining rigorous data curation with DL models. GinDB encompasses 753 ginsenosides, systematically categorized into structural subtypes and botanical origins, revealing clear discovery trends and taxonomic distributions. To overcome the critical lack of annotated physicochemical and biological data, we developed and integrated DL models capable of accurately predicting CCS, t_R_, and biological activity based on molecular features. To ensure broad accessibility, we deployed these predictive tools through a publicly available web platform (https://balalab-skku.org/GinDB-AI/), equipped with user-friendly modules for data submission, compound browsing, and result retrieval. With extensive documentation and downloadable datasets, GinDB-AI supports reproducible, large-scale computational investigations. Altogether, GinDB-AI is more than just a curated database; it is an AI-driven workbench that empowers researchers to rapidly annotate uncharacterized ginsenosides and predict their key molecular properties. By offering a scalable framework, this platform is well-positioned to accelerate discovery in ginseng pharmacology, natural product chemistry, and drug development, guiding experimental prioritization and uncovering novel bioactive candidates.

## Author contributions

**Nguyen Doan Hieu Nguyen**: Conceptualization, Methodology, Software, Validation, Visualization, Writing - original draft, Writing - review & editing. **Vinoth Kumar Sangaraju**: Conceptualization, Methodology, Software, Validation, Visualization, Writing - original draft, Writing - review & editing. **Duong Thanh Tran**: Conceptualization, Methodology, Software, Validation, Writing - original draft, Writing - review & editing. **Nhat Truong Pham**: Conceptualization, Methodology, Software, Validation, Writing - original draft, Writing - review & editing. **Jae Youl Cho**: Writing - original draft, Supervision, Investigation. **Balachandran Manavalan**: Conceptualization, Writing - review & editing, Writing - original draft, Supervision, Investigation, Funding acquisition.

## Declaration of generative AI and AI-assisted technologies in the writing process

During the preparation of this work, the authors used ChatGPT 4.0 to make the sentence more fluent and easier to understand. After using this tool/service, the authors reviewed and edited the content as needed and took full responsibility for the content of the published article.

## Funding

This work was supported by a grant from the 10.13039/501100015826Korean Society of Ginseng, Seoul, Korea, received in 2023. It was also supported by the Department of Integrative Biotechnology, Sungkyunkwan University (SKKU), and the BK21 FOUR Project.

## Declaration of competing interest

The authors declare no competing interests.

## Data Availability

The database can be accessed and downloaded at https://balalab-skku.org/GinDB-AI/. The tools for predicting physicochemical values and pharmacological potential of ginseng are provided at https://balalab-skku.org/GinDB-AI/tools/. The implementation of the model has been made publicly available at https://github.com/cbbl-skku-org/GinDB-AI/along with the pretrained weights.

## Footnotes

2 https://go.drugbank.com/.

3 https://www.ebi.ac.uk/chebi/.

4 https://pubchem.ncbi.nlm.nih.gov/.

5 https://www.ebi.ac.uk/chembl/.

6 https://revvitysignals.com/products/research/chemdraw.

7 https://pypi.org/project/rdkit/.
