# Supplementary material for: GinDB-AI: An integrated ginsenoside database and AI-driven platform for multidimensional information and biological activity prediction
Source: J Ginseng Res. 2026 Feb 4;50(3):100986. doi: 10.1016/j.jgr.2026.100986 (PMC13149897; doi:10.1016/j.jgr.2026.100986)
Supplement: Multimedia component 1 [file mmc1.docx]

**GinDB-AI: An integrated ginsenoside database and AI-driven platform for multidimensional information and biological activity prediction**

Nguyen Doan Hieu Nguyen^a,1^, Vinoth Kumar Sangaraju^a,1^, Duong Thanh Tran^a^, Nhat Truong Pham^a^, Balachandran Manavalan^a,*^

^a^Department of Integrative Biotechnology, College of Biotechnology and Bioengineering, Sungkyunkwan University, Suwon, 16419, Gyeonggi-do, Republic of Korea

^1^These authors contributed equally to this work and shared first authorship.

*Corresponding author at:

Department of Integrative Biotechnology, College of Biotechnology & Bioengineering, Sungkyunkwan University

E-mail address: [[bala2022@skku.edu](mailto:bala2022@skku.edu)](mailto:bala2022@skku.edu)

Phone: +82-31-299-4858

Fax: +82-31-290-7870

**Supplementary Texts**

**Text S1. Dataset preparation for AI-based prediction tools**

Each sample in the dataset for the regression tasks was represented by its canonical SMILES (Weininger, 1988) string and included values for CCS, t_R_, and mass-to-charge ratio (m/z). To account for ionization states, we also included adduct information, specifically [M−H]⁻ (deprotonated) and [M−H+HCOOH]⁻ (formate adduct). We focused our predictive modeling efforts on t_R_ and CCS, intentionally excluding the prediction of m/z. The m/z value can be calculated precisely from a molecule's formula and charge, making an artificial intelligence (AI)-based model unnecessary. In contrast, t_R_ and CCS are complex physicochemical properties that depend on molecular structure, conformation, and experimental conditions, making them ideal targets for data-driven AI approaches. To ensure data integrity, we standardized the dataset by removing redundant samples, retaining only one entry per unique SMILES string. This reduced the dataset to 101 unique samples and mitigated potential bias from isomers, which share a molecular formula but have distinct structures. To manage the skewness and influence of outliers in the target variables, we applied a logarithmic transformation to the t_R_ and CCS values. Finally, we split the dataset into a training set (90 samples) and a testing set (11 samples) using a 9:1 ratio. The adduct label reduces potential bias from redundant samples; we retained only one entry per unique SMILES input, which resulted in 101 unique samples. This step also addresses the issue of including isomers, as their presence can bias models that rely on molecular descriptors. To mitigate the effect of outliers and distribution skewness in our target values (t_R_ and CCS), we applied a logarithmic transformation to the target values. The final dataset was then partitioned into training (90 samples) and testing (11 samples) sets.

**Text S2. Dataset preparation for AI-based biological activities prediction tool**

The dataset for the classification task contained 527 ginsenoside samples with available bioactivity data. During preprocessing, we removed eight duplicate samples (three active and five inactive), resulting in a final set of 519 unique samples. Among these, 257 samples were reported to exhibit one or more biological activities (positive class), while the remaining 262 samples showed no reported activity (negative class). The dataset was divided into training and testing sets using a stratified split to ensure a balanced representation of positive and negative classes. An 8:2 ratio was applied, resulting in a training set of 415 samples (205 positive and 210 negative) and a testing set of 104 samples (52 positive, 52 negative).

**Text S3. Feature extraction for prediction tools**

To enable the application of AI algorithms, we converted the canonical SMILES strings into a comprehensive numerical feature space, incorporating both conventional molecular descriptors and advanced, pre-trained molecular embeddings. First, we extracted a set of molecular descriptors (MDs) and molecular fingerprints (MFs) to encode the traditional physicochemical properties and structural motifs of the ginsenosides. We employed RDKit to generate 12 distinct feature sets: one MD (E-State) [1] and 11 MFs, including Avalon [2], Extended [3], FP2 [4], Hybridization [3], Morgan fingerprints (including ECFP4, ECFP6, FCFP4, and FCFP6) [5], PubChem [6], Standard [3], and RDKit fingerprints [7]. These representations are well-established in cheminformatics and are effective for capturing the fundamental chemical characteristics of molecules.

Second, to capture the deep characteristics and semantic relationships within the molecular structures, we leveraged pretrained biological NLP-based models. We selected seven state-of-the-art pretrained models that transform molecular strings into high-dimensional embedding vectors: MolT5 (small, base, and large) [8], SciBERT [9], ChemBERTa [10], SELFormer [11], and Mol2Vec [12]. Notably, these models, pre-trained on large-scale molecular strings, are designed to capture complex, contextual information that is often missed by conventional feature descriptors. While most of our selected models were trained on SMILES, the SELFormer model was specifically trained on the SELFIES molecular format. Thus, we converted our SMILES strings to SELFIES using the *selfies*^[[1]](#footnote-1)^ package prior to the feature extraction with the SELFormer model. Detailed descriptions of the features used and their processing pipelines were provided in Table S2.

**Text S4. Development of regression models**

We proposed DL-based models for identifying multidimensional analytical properties (regression tasks) with two consecutive modules: Feature Transform (FT) and Regression (Fig. S1D). This modular design allows for the independent processing and integration of diverse feature types. Each module was designed with specific functions, implementation methodologies, and comprehensive training protocols discussed below.

**Feature Transform module:** The FT module is responsible for converting the raw input features (molecular descriptors, fingerprints, or NLP embeddings) into a unified latent space representation. This module consists of a series of fully connected (FC) layers that map the input feature vector into a condensed vector, denoted as $f_{1}$. Previous studies have demonstrated that polarizability or adduct information plays an important role in the accurate prediction of CCS [13,14,15]. To integrate this information, we constructed a one-hot vector to represent the two adduct types in our dataset, [M−H]⁻ and [M−H+HCOOH]⁻. Materials and methods. This vector was passed through an FC layer to generate a corresponding embedding, denoted as $f_{2}$. The two embeddings $f_{1}$ and $f_{2}$, were then concatenated to form $f_{concat}$, which serves as the final input for the subsequent Regression module.

**Regression module:** The module receives the transformed features ($f_{1}$ or $f_{concat}$) from the FT module and performs the final prediction for each target variable (t_R_ and CCS). It is composed of a sequence of FC layers, Normalization, Activation, and Dropout layers. FC layers enable the model to learn complex, nonlinear mappings between the molecular representations and the target values. Normalization layers stabilize and accelerate model training by mitigating internal covariate shifts. Activation layers introduce nonlinearity, allowing the model to capture complex relationships between input features and outputs. Dropout layers serve as a regularization technique, randomly deactivating a subset of neurons during training to prevent overfitting and enhance the model’s generalization capabilities on unseen data.

**Text S5. Development of the classification model**

To address the classification task of determining whether ginsenosides exhibit biological activity, we designed an architecture consisting of two modules: Feature Transform and Classification. The overall architecture is detailed as follows:

**Feature Transform module:** In this module, the input feature $f\in\mathbb{R}^{d}$ is processed through $N$ sequential blocks, each comprising three core layers: FC, normalization, and activation.

The FC layer projects the features into a specific intermediate dimension to allow for nonlinear transformations. The Normalization layer stabilizes the training by mitigating internal covariate shift, and the activation introduces non-linearity while retaining smoothness, enabling the model to better capture complex patterns. In each block, these layers are applied in sequence. The dimensionality of the features is gradually reduced from $d$ to $\left\lfloor\frac{d}{N+1} \right\rfloor$ in uniform steps, promoting compact and hierarchical feature representation. The transformation process can be summarized as follows:

$$\begin{aligned} f^{\left( k \right)}=\text{Activation}\left( \text{Norm}\left( \text{FC}\left( f^{\left( k-1 \right)} \right) \right) \right), \text{where} f^{\left( 0 \right)}=f\in\mathbb{R}^{d}\#\left( 1 \right) \end{aligned}$$

After each transformation step $k$, the feature dimensionality is reduced as:

$$\begin{aligned} f^{\left( k \right)}\in\mathbb{R}^{\left\lfloor\frac{d}{k+1} \right\rfloor}, \mathrm{for} k=1,2,\ldots,N\#\left( 2 \right) \end{aligned}$$

The final representation $f^{(N)}$ at the last block will be processed by the next module, which is detailly mentioned in the next section.

**Classification module:** In this module, the output feature $f^{(N)}$ is passed through an FC layer followed by a softmax activation function to produce the probability distribution over the two classes. This process can be summarized as:

$$\begin{aligned} p=\text{softmax}\left( \text{FC}\left( f^{\left( N \right)} \right) \right)\#\left( 3 \right) \end{aligned}$$

Where $p\in\mathbb{R}^{2}$ represents the predicted probabilities for the two classes.

**Text S6. Implementation details**

To determine the best hyperparameters to train the models while avoiding risks of bias and data leakage, we comprehensively employed 10-fold cross-validation and the grid search mechanism. After identifying the optimal configuration yielding the highest training performance, we applied it to train the final models on the entire training set. We used the AdamW optimizer [16] with a scheduler to decay the learning rate of each parameter group by multiplying every specific number of steps by a factor. The training processes are carried out by the Smooth L1 [17] and Focal [18] losses for the regression and classification, respectively.

In the classification task, we employed Focal loss (FL) instead of binary cross-entropy loss to control class weighting. Although the dataset is balanced, we hypothesize that one class may be inherently more difficult to learn, requiring greater emphasis during training. The FL is implemented as follows:

$$\begin{aligned} \text{FL}\left( p_{t} \right)=-\alpha_{t}\left( 1-p_{t} \right)^{\beta}\log\left( p_{t} \right)\#\left( 15 \right) \end{aligned}$$

where $p_{t}$ is the predicted probability of the true class ($p_{t}=p$ if the label is 1, and $p_{t}=1-p$ if the label is 0), $\alpha_{t}$ is a balancing factor for the class weight, $\beta$ is a modulating factor that reduces the loss for well-classified examples. The details of the hyperparameter search range can be found in Table S4.

**Text S7. Evaluation metrics**

**Regression:** To comprehensively measure the effectiveness of the regression models, we employed three popular metrics: mean absolute error (MAE), mean squared error, and coefficient of determination (R^2^). Their formulas are defined below:

$$\begin{aligned} MAE=\frac{1}{n}\sum|y_{i}-\hat{y_{i}}|\#\left( 4 \right) \end{aligned}$$

$$\begin{aligned} MSE=\frac{1}{n}\sum\left( y_{i}-\hat{y_{i}} \right)^{2}\#\left( 5 \right) \end{aligned}$$

$$\begin{aligned} R^{2}=1-\frac{SSR}{SST}=\frac{\sum\left( y_{i}-\hat{y_{i}} \right)^{2}}{\sum\left( y_{i}-\bar{y} \right)^{2}}\#\left( 6 \right) \end{aligned}$$

Where $y_{i}$ and $\hat{y_{i}}$ denote the ground truth and predicted values of the sample at the index $i$, and $n$ represents the total number of samples. $SSR$ and $SST$ stand for Sum of Squared Residuals, which is the sum of the squared differences between the actual and predicted values, and Total Sum of Squares, which is the sum of the squared differences between the actual values and the mean of the actual values. $\bar{y}$ denotes the mean of the actual values.

**Classification:** To evaluate the performance of classification models, we use five well-known metrics, including F1 score (F1), accuracy (ACC), sensitivity (Sn), specificity (Sp), and Matthews correlation coefficient (MCC) as follows:

$$\begin{aligned} F1=\frac{TP}{2TP+FN+FP}\#\left( 7 \right) \end{aligned}$$

$$\begin{aligned} ACC=\frac{TP+TN}{TP+TN+FP+FN}\#\left( 8 \right) \end{aligned}$$

$$\begin{aligned} Sn=\frac{TP}{TP+FN}\#\left( 9 \right) \end{aligned}$$

$$\begin{aligned} Sp=\frac{TN}{TN+FP}\#\left( 10 \right) \end{aligned}$$

$$\begin{aligned} MCC=\frac{TP\times TN-FP\times FN}{\sqrt{\left( TP+FP \right)\left( TP+FN \right)\left( TN+FP \right)\left( TN+FN \right)}} \#\left( 11 \right) \end{aligned}$$

TP, TN, FP, and FN denote true positives, true negatives, false positives, and false negatives, respectively. F1 is the harmonic mean of precision and recall. ACC measures the overall correctness of predictions. Sn is the proportion of correctly identified positive instances, while Sp is the proportion of correctly identified negative instances. MCC provides a balanced evaluation using all four confusion matrix terms. Among these metrics, we chose MCC as the main metric to evaluate overall performance during the training and identify the optimal configuration.

Additionally, we utilized the area under the receiver operating characteristic curve (AUC), which reflects the model’s ability to distinguish between classes across thresholds. The formula for AUC is as follows:

$$\begin{aligned} AUC=\sum_{i=1}^{T} \left( FPR_{i+1}-FPR_{i} \right)\cdot\frac{TPR_{i}++TPR_{i+1}}{2},\#\left( 12 \right) \end{aligned}$$

$$\begin{aligned} FPR_{i}=\frac{FP_{i}}{FP_{i}+TN_{i}},\#\left( 13 \right) \end{aligned}$$

$$\begin{aligned} TPR_{i}=\frac{TP_{i}}{TP_{i}+FN_{i}}.\#\left( 14 \right) \end{aligned}$$

Here, while the FP rate ($FPR_{i}$) defines the proportion of negative samples incorrectly classified, TP rate ($TPR_{i}$) quantifies the proportion of positive samples correctly classified at the threshold $i$, and $T$ denotes the total number of thresholds evaluated.

**Text S8. Performance assessment of regression models**

**Collision cross section**

To rigorously assess the predictive performance and robustness of our proposed DL model across various molecular features, we compared the top five configurations with the highest training R^2^ scores (Fig. S2A, *Without adduct* section). Interestingly, the model with Mol2Vec feature achieved the highest training R^2^ score of 0.9337, representing an improvement of 0.56% to 2.3569% compared to other models, while reducing MAE and MSE of 0.0031 and 0.0003, respectively. Notably, models leveraging pretrained NLP embeddings, such as Mol2Vec, MolT5-base, SciBERT, and MolT5-small, show superior performance in the five, underscoring the effectiveness of NLP-based molecular encodings for feature extraction. Evaluation of these models on the independent dataset showed strong generalization capability. Specifically, the Mol2Vec-based model remained the top performer, achieving MAE, MSE, and R2 scores of 0.0138, 0.0003, and 0.9343, respectively.

We then examined the performance of our proposed framework by incorporating adduct information, acknowledging that they influence CCS due to their impact on ion mobility (Fig. S2A, *With adduct* section). Interestingly, among the top five highest training R^2^ configurations, the model utilizing the Mol2Vec-based model again achieved the highest performance, with MAE reductions of 0.0006-0.0035, MSE reduction up to 0.0002, and R^2^ increases of 0.9140%-3.0187% compared to other models. Upon independent testing, Mol2vec achieved MAE, MSE, and R^2^ of 0.0147, 0.0003, and 0.9143, respectively. Compared to the best model without adduct information, the adduct-inclusive model improved training R^2^ performance by 0.5141%, while slightly reducing the testing performance by 2.1406% (Fig. S2A). Nevertheless, its superior training accuracy, combined with its competitive testing performance, supports its selection as the final optimal model for CCS prediction in ginsenosides.

**Retention time**

We first evaluated the predictive capability of the model’s performance in predicting t_R_ of ginsenosides based solely on molecular features. To comprehensively assess model performance, we analyzed the top five models based on training R^2^ scores. Among these, models using EState and MolT5-based features demonstrated superior performance, with training R^2^ above 0.74, while the remaining models achieved R^2^ below 0.70. Specifically, the model incorporating EState descriptor achieved the best performance, with improvements ranging from 1.7349%-13.7492% in R^2^ score, and corresponding reductions in MAE (0.0133-0.1923) and MSE (0.0106-0.1305) relative to other models. Although EState, which is an MF, showed an impressive training performance, the other four positions in the top five - namely MolT5-base, SciBERT, MolT5-small, and Mol2Vec - are NLP features. These results not only presented a high correlation between the EState feature and t_R_ value, but also demonstrated the potential of the NLP features in t_R_ prediction. Based on this analysis, the model trained on Estate was selected as the optimal model for t_R_ prediction. Evaluation on the independent test dataset revealed high consistency and transferability. The Estate model achieved an MAE of 0.1605, an MSE of 0.0348, and an R^2^ score of 0.8774. Notably, the testing R^2^ score improved by 17.5824% compared to the corresponding training performance, indicating excellent model transferability.

Despite the absence of experimental studies on the relationship between adduct formation and t_R_, we explored whether including adduct information could enhance prediction accuracy. Interestingly, integration of adduct information negatively impacted model performance. The best model with adducts exhibited a 1.769% decrease in training R^2^ and a 9.6307% reduction in testing R^2^ compared to the corresponding model without adduct information. These results suggest that adducts are not correlated with retention time, and their inclusion introduces noise rather than an informative signal. We observed that the model excluding adducts showed tighter convergence to the diagonal in the prediction vs. observation plot-indicative of improved predictive alignment (Fig. S2B). Consequently, we conclude that excluding adduct information yields more accurate and stable predictions of t_R_, and therefore opted not to incorporate it in the final model.

**Text S9. Performance assessment of biological activity classification model**

To assess the effectiveness of classifying the biological activity in ginsenosides, we constructed and evaluated our proposed models using 19 molecular feature representations. Detailed performance results across all model-feature combinations are provided in Tables S9 and S10.

Fig. S3 summarizes the top five performing models for the models, ranked by MCC on the training dataset. Among the models, the model using Mol2Vec features achieved the highest overall performance, highlighting the synergy between pretrained molecular embedding and the DL architecture. The models primarily utilize embedding-based features from pre-trained models, which encode richer, high-dimensional molecular characteristics. Among these, Mol2Vec features emerged as the most frequently selected feature across top configurations, underscoring the effectiveness of this feature in capturing biologically relevant information.

While some models exhibited reduced generalization to the test set (Fig. S3), particularly those features using MolT5_large, MolT5_small, RDKit, and MolT5_base, the model with Mol2Vec maintained excellent transferability, achieving MCC = 0.3464, ACC = 0.6731, F1 = 0.6792, and AUC = 0.7245. Compared to other models, the DL-Mol2Vec demonstrated improvements of 3.50%-32.70% in MCC, 1.93%-16.35% in ACC, 2.27%-18.42% in F1, and 4.27%-10.97% in AUC, highlighting its robust generalization capability. Collectively, these results demonstrate that our proposed model, combined with the Mol2Vec feature, demonstrates both high predictive performance and robust generalization, making it the ideal choice for deployment in biological activity classification of ginsenosides. This combination effectively leverages the strengths of DL while capturing meaningful molecular representations, providing a reliable framework for future applications.

**Supplementary Tables**

Table S1. List of molecular formula and weight corrections for misreported compounds in existing databases and literature

|  |  | **Molecular Formula** | | **Molecular Weight** | |  |
| --- | --- | --- | --- | --- | --- | --- |
| **No.** | **Compound Name** | **Reported** | **Corrected** | **Reported** | **Corrected** | **References** |
| 1 | Chikusetsusaponin FK6 | C₅₃H₈₈O₂₂ | C₅₃H₉₀O₂₂ | 1077.234 | 1079.281 | [19] |
| 2 | Ginsenoside Ra5 | C₆₀H₉₉O₂₇ | C₆₀H₁₀₀O₂₇ | 1252.392 | 1253.433 |  |
| 3 | Ginsenoside Ra7 | C₅₇H₉₃O₂₃ | C₅₇H₉₄O₂₃ | 1146.314 | 1147.356 |  |
| 4 | Ginsenoside Ra1 | C₅₈H₉₈O₂₅ | C₅₈H₉₈O₂₆ | 1195.364 | 1211.396 |  |
| 5 | Pseudoginsenoside Rt3 | C₄₀H₆₈O₁₃ | C₄₁H₇₀O₁₃ | 756.944 | 770.998 |  |
| 6 | Saponin Ve | C₄₇H₇₄O₁₈ | C₄₇H₇₆O₁₇ | 927.062 | 913.108 |  |
| 7 | Ginsenoside Rs4 | C₄₄H₇₂O₁₃ | C₄₄H₇₂O₁₄ | 809.016 | 825.046 |  |
| 8 | Ginsenoside Rs6 | C₃₈H₆₀O₈ | C₃₆H₆₀O₈ | 644.86 | 620.868 |  |
| 9 | Floralginsenoside H | C₅₀H₈₄O₂₁ | C₅₀H₈₄O₂₂ | 1021.172 | 1037.2 |  |
| 10 | Ginsenoside Rs5 | C₄₄H₇₂O₁₃ | C₄₄H₇₂O₁₄ | 809.016 | 825.046 |  |
| 11 | Ginsenoside Rh6 | C₃₆H₆₂O₁₁ | C₃₆H₆₂O₁₀ | 670.856 | 654.882 |  |
| 12 | Ginsenoside Rg5 | C₄₂H₇₀O₁₂ | C₄₂H₇₀O₁₃ | 766.98 | 783.009 |  |
| 13 | Vinaginsenoside R20 | C₄₈H₈₀O₁₉ | C₄₈H₈₀O₂₀ | 961.12 | 977.148 |  |
| 14 | Chikusetsusaponin FK2 | C₄₈H₈₀O₁₈ | C₄₈H₈₀O₁₉ | 945.12 | 961.149 |  |
| 15 | Chikusetsusaponin FK3 | C₅₃H₈₈O₂₂ | C₅₃H₈₈O₂₃ | 1077.234 | 1093.264 |  |
| 16 | Gypenoside CIII | C₄₇H₈₀O₁₉Na | C₄₇H₈₀O₁₉ | 972.1 | 949.138 | [20] |
| 17 | Gypenoside CIV | C₄₂H₇₂O₁₅Na | C₄₂H₇₂O₁₅ | 839.986 | 817.023 |  |
| 18 | Gypenoside CV | C₄₇H₈₀O₁₈Na | C₄₇H₈₀O₁₈ | 956.1 | 933.139 |  |
| 19 | Gypenoside CVI | C₅₂H₈₈O₂₂Na | C₅₂H₈₈O₂₂ | 1088.214 | 1065.254 |  |
| 20 | Gypenoside CVII | C₅₂H₈₈O₂₁Na | C₅₂H₈₈O₂₁ | 1072.214 | 1049.255 |  |
| 21 | Gypenoside CVIII | C₅₂H₈₈O₂₂Na | C₅₂H₈₈O₂₂ | 1088.214 | 1065.254 |  |
| 22 | Gypenoside CIX | C₄₈H₈₂O₂₀Na | C₄₈H₈₂O₂₀ | 1002.126 | 979.164 |  |
| 23 | Gypenoside CX | C₄₇H₇₈O₁₈Na | C₄₇H₇₈O₁₈ | 954.084 | 931.123 |  |
| 24 | Gypenoside CXI | C₅₃H₉₀O₂₄Na | C₅₃H₉₀O₂₄ | 1134.24 | 1111.279 |  |
| 25 | Gypenoside CXII | C₅₃H₉₀O₂₄Na | C₅₃H₉₀O₂₄ | 1134.24 | 1111.279 |  |
| 26 | Gypenoside CXIII | C₄₇H₇₉O₁₉ | C₄₇H₈₀O₁₉ | 948.102 | 949.138 |  |
| 27 | Gypenoside CXIV | C₄₈H₈₂O₂₀Na | C₄₈H₈₂O₂₀ | 1002.126 | 979.164 |  |
| 28 | Gypenoside CXV | C₄₇H₈₀O₁₈Na | C₄₇H₈₀O₁₈ | 956.1 | 933.139 |  |
| 29 | Notoginsenoside SFt5 | C₃₆H₆₂O₉Na | C₃₆H₆₂O₉ | 661.846 | 638.883 | [21] |
| 30 | Notoginsenoside SFt6 | C₃₆H₆₁O₈ | C₃₆H₆₀O₈ | 621.848 | 620.868 |  |
| 31 | Notoginsenoside SFt7 | C₃₆H₆₁O₈ | C₃₆H₆₀O₈ | 621.848 | 620.868 |  |
| 32 | Notoginsenoside SFt8 | C₃₆H₆₁O₈ | C₃₆H₆₀O₈ | 621.848 | 620.868 |  |
| 33 | Notoginsenoside SFt9 | C₄₇H₈₀O₁₉Na | C₄₇H₈₀O₁₉ | 972.1 | 949.138 |  |
| 34 | Notoginsenoside SFt10 | C₄₇H₇₈O₁₆Na | C₄₇H₇₈O₁₆ | 922.084 | 899.125 |  |
| 35 | notoginsenoside ST6 | C₃₅H₅₇O₉ | C₃₅H₅₆O₉ | 621.806 | 620.824 | [22] |
| 36 | notoginsenoside ST7 | C₃₅H₅₇O₉ | C₃₅H₅₆O₉ | 621.806 | 620.824 |  |
| 37 | Epoxynotoginsenoside A | C₅₅H₉₃O₂₃ | C₅₄H₉₀O₂₃ | 1122.294 | 1107.291 | [23] |
| 38 | Ginsenoside Rg10 | C₄₂H₆₉O₁₃ | C₄₂H₇₀O₁₃ | 781.972 | 783.009 |  |
| 39 | Hemsloside G1 | C₅₃H₈₃O₂₃ | C₅₃H₈₄O₂₃ | 1088.194 | 1089.232 |  |
| 40 | Kaempferol-3-O-(6-O-Ma-D-glucopyranoside) | C₃₀H₃₁O₁₉ | C₃₀H₃₂O₁₉ | 695.548 | 696.567 |  |
| 41 | Kaempferol-3-O-(6-O-Ma-D-glucopyranoside),7-O-[-D-Glucopyranosyl-(1-2)-L-rhamnopyranoside] | C₃₆H₄₁O₂₃ | C₃₆H₄₂O₂₃ | 841.688 | 842.709 |  |
| 42 | Kaempferol-3-O-[-D-Glucopyranosyl-(1→2)-L-rhamnopyranoside],7-O-(6-O-malonyl-D-glucopyranoside) | C₃₆H₄₁O₂₃ | C₃₆H₄₂O₂₃ | 841.688 | 842.709 |  |
| 43 | Koryoginsenoside-R1 | C₄₂H₆₈O₁₃ | C₄₂H₆₈O₁₃ | 780.964 | 780.99 |  |
| 44 | Lutonarin | C₅₅H₇₇O₁₅ | C₂₇H₃₀O₁₆ | 978.166 | 610.521 |  |
| 45 | Ma-Rutin | C₃₀H₃₁O₁₉ | C₃₀H₃₂O₁₉ | 696.567 | 696.567 |  |
| 46 | Rutin | C₂₇H₂₉O₁₆ | C₂₇H₃₀O₁₆ | 609.502 | 610.521 |  |
| 47 | Vinaginsenoside R15 | C₄₂H₇₂O₁₃ | C₄₂H₇₂ O₁₅ | 784.996 | 817.023 |  |
| 48 | Yesanchinoside D | C₄₄H₇₄O₁₅ | C₄₄H₇₄O₁₆ | 843.032 | 859.06 |  |

Table S2. Table showing the 19 feature encodings and their dimensions utilized in DL models for regression and classification.

| **Feature Types** | **Feature Encodings** | **Feature Dimensions** |
| --- | --- | --- |
| **NLP-based Features** | MolT5-small | 512D |
|  | MolT5-base | 768D |
|  | MolT5-large | 1024D |
|  | SciBERT | 768D |
|  | ChemBERTa | 768D |
|  | SELFormer | 768D |
|  | Mol2Vec | 300D |
| **Molecular Fingerprints** | Avalon | 512D |
|  | Extended | 1024D |
|  | FP2 | 1024D |
|  | Hybridization | 1024D |
|  | Morgan ECFP4 | 2048D |
|  | Morgan ECFP6 | 2048D |
|  | Morgan FCFP4 | 2048D |
|  | Morgan FCFP6 | 2048D |
|  | PubChem | 881D |
|  | Standard | 1024D |
|  | RDKit | 2048D |
| **Molecular Descriptor** | E-State | 245D |

Table S3. Dataset distribution across regression and classification tasks

| **Type** | **Dataset Split** | **Number of Samples** |
| --- | --- | --- |
| **Regression** | Training | 90 |
|  | Testing | 11 |
| **Classification** | Training | 415 |
|  | Testing | 104 |

Table S4. Configurations in the grid search applied in the k-fold cross-validation process.

| **Hyperparameter** | **Values** |
| --- | --- |
| Normalization layer | Batch Normalization, Layer Normalization |
| Activation layer | ReLU, Leaky ReLU, GELU |
| Dropout rate | 0.0, 0.05, 0.1 |
| Hidden dimension | 16, 32, 64 |
| Learning rate | 0.01, 0.001, 0.0001 |
| Number of epochs | 100, 300, 500 |
| Batch size | 8, 16, 32 |

**Supplementary Figures**

**
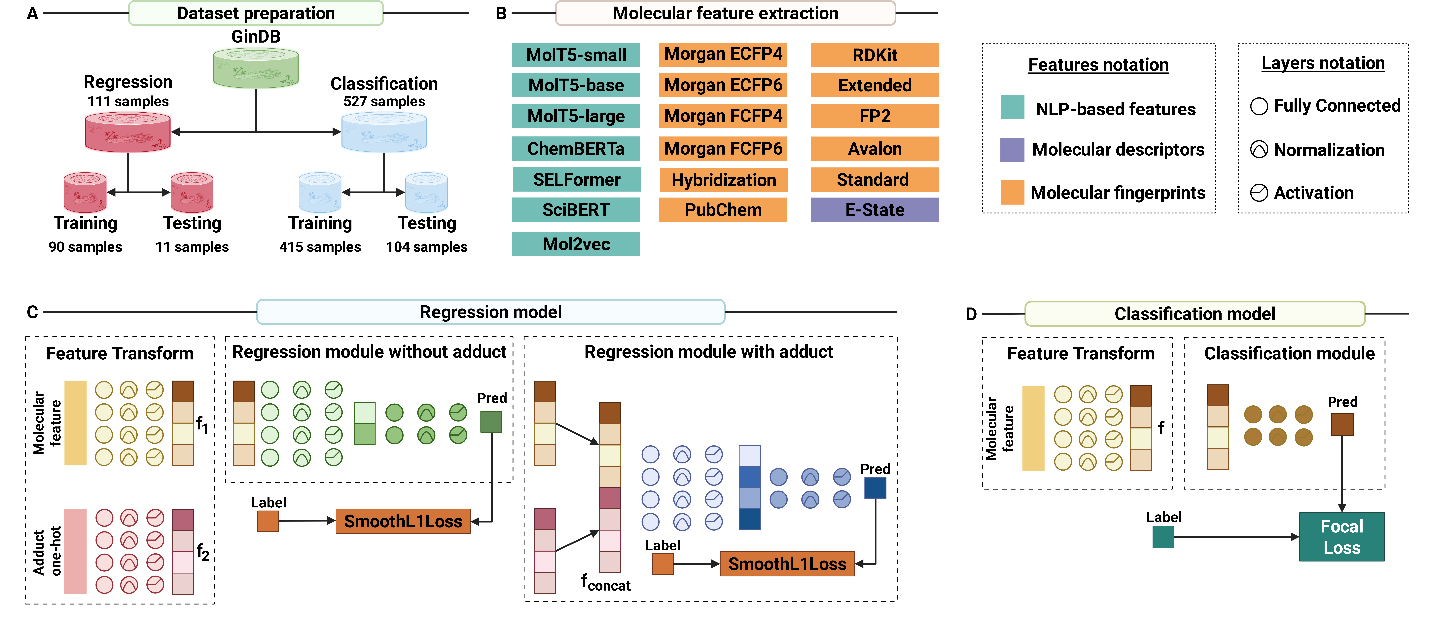
**

**Fig. S1.** The comprehensive workflow for developing deep learning models aimed at predicting physicochemical properties and pharmacological potential of ginsenosides. (A) The dataset preparation phase involving preprocessing the data for each specific task and subsequently splitting it into training and testing sets. (B) The extraction of molecular features from the input molecular strings using NLP-based pretrained models, molecular descriptors, and molecular fingerprints. (C) Our proposed deep learning architecture for predicting physicochemical values designed to train each target independently. (D) Our proposed deep learning architecture for predicting the pharmacological potential of ginsenoside.


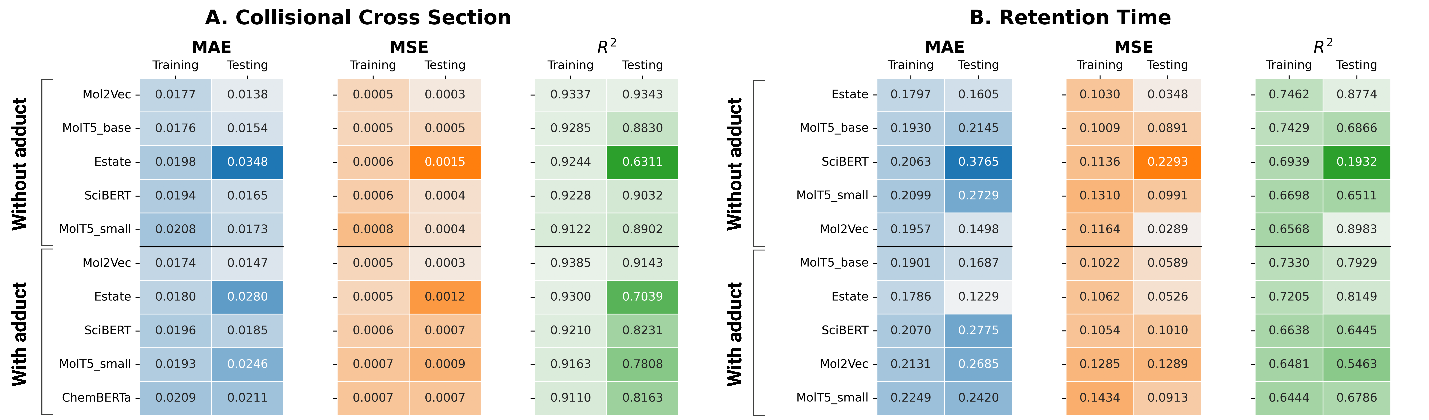


**Fig. S2.** The training and testing performance of the models in predicting physicochemical properties reported using three metrics: MSE, MAE and R^2^. Each subfigure is divided into two sections, showcasing the performance of the proposed model and the ablation study of the adduct information. For each approach, we present the top five models with the highest training R2 score and their corresponding performance. (A) The performance of the models in predicting collisional cross section. (B) The performance of the models in predicting retention time.

**
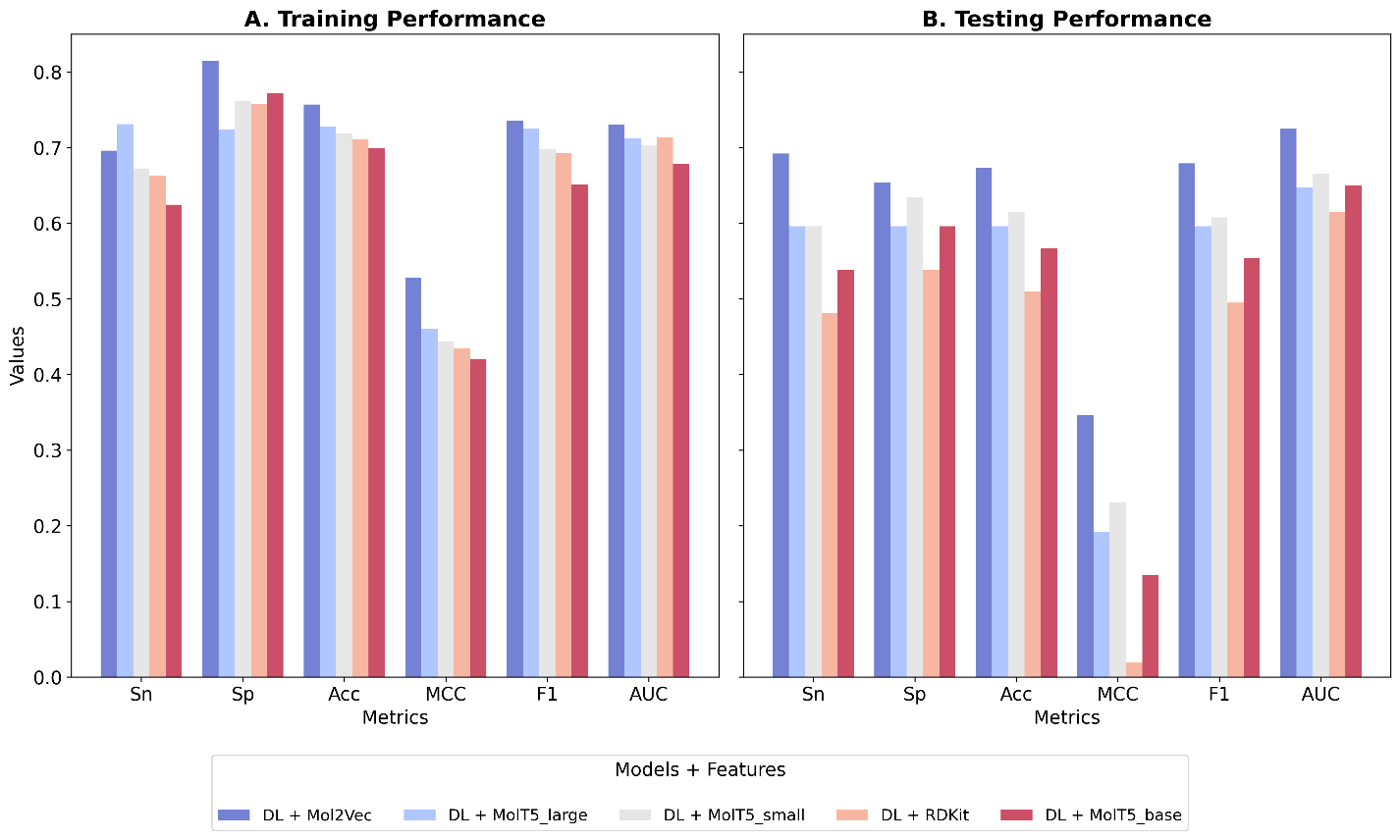
**

**Fig. S3.** The training and testing performance of the models in predicting the pharmacological potential of ginsenoside reported using six metrics: Sn, Sp, Acc, MCC, F1, and AUC. For each approach, we present the top five models with the highest training MCC scores and their corresponding performance. (A) The training performance of the models. (B) The testing performance of the models.


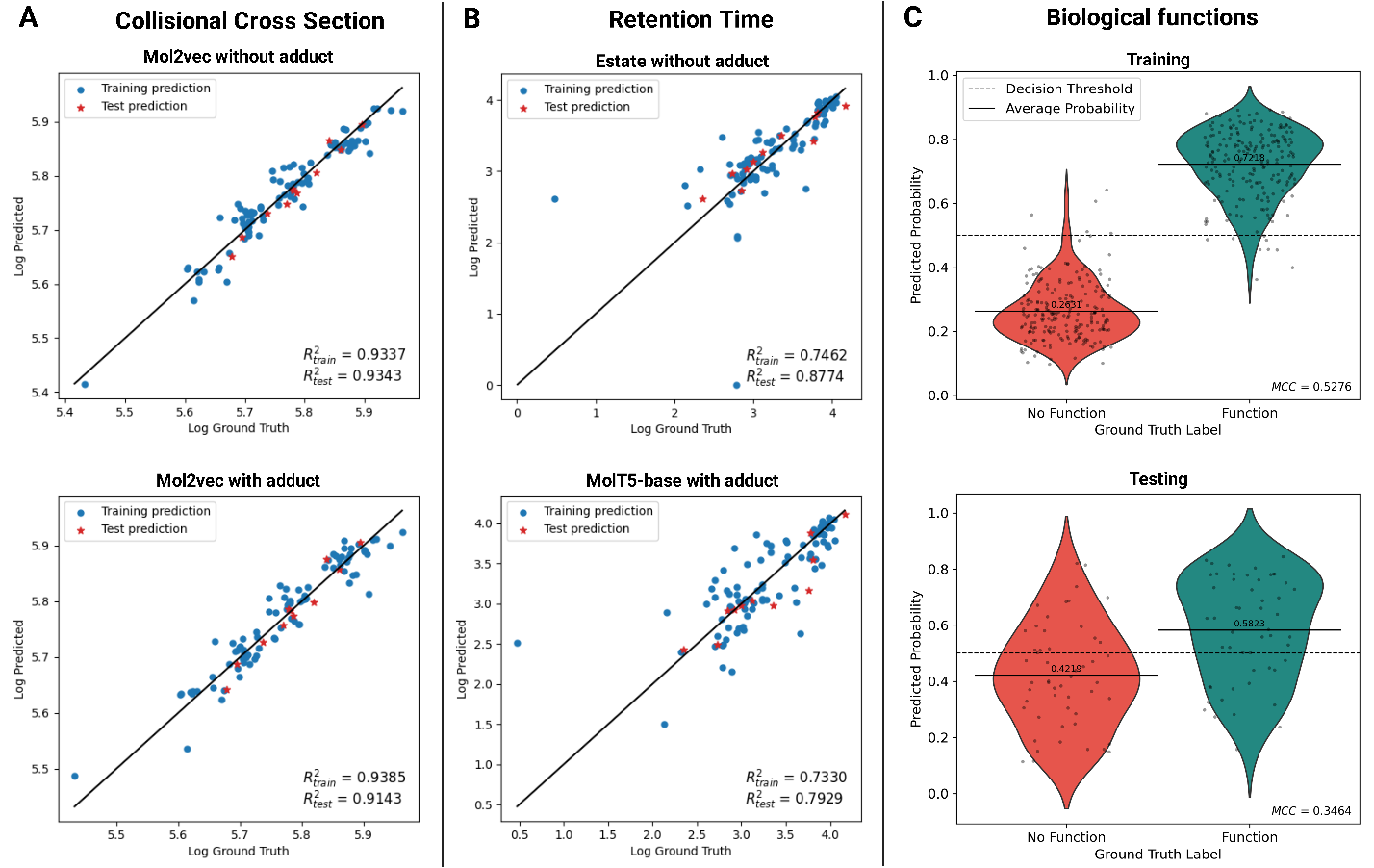


**Fig. S4.** Predictions of the optimal models on the testing set in each task. (A) Prediction of the regression models in predicting collisional cross-section. (B) Prediction of the regression models in predicting retention time. (C) Prediction of the classification models in predicting the pharmacological potential of ginsenoside.


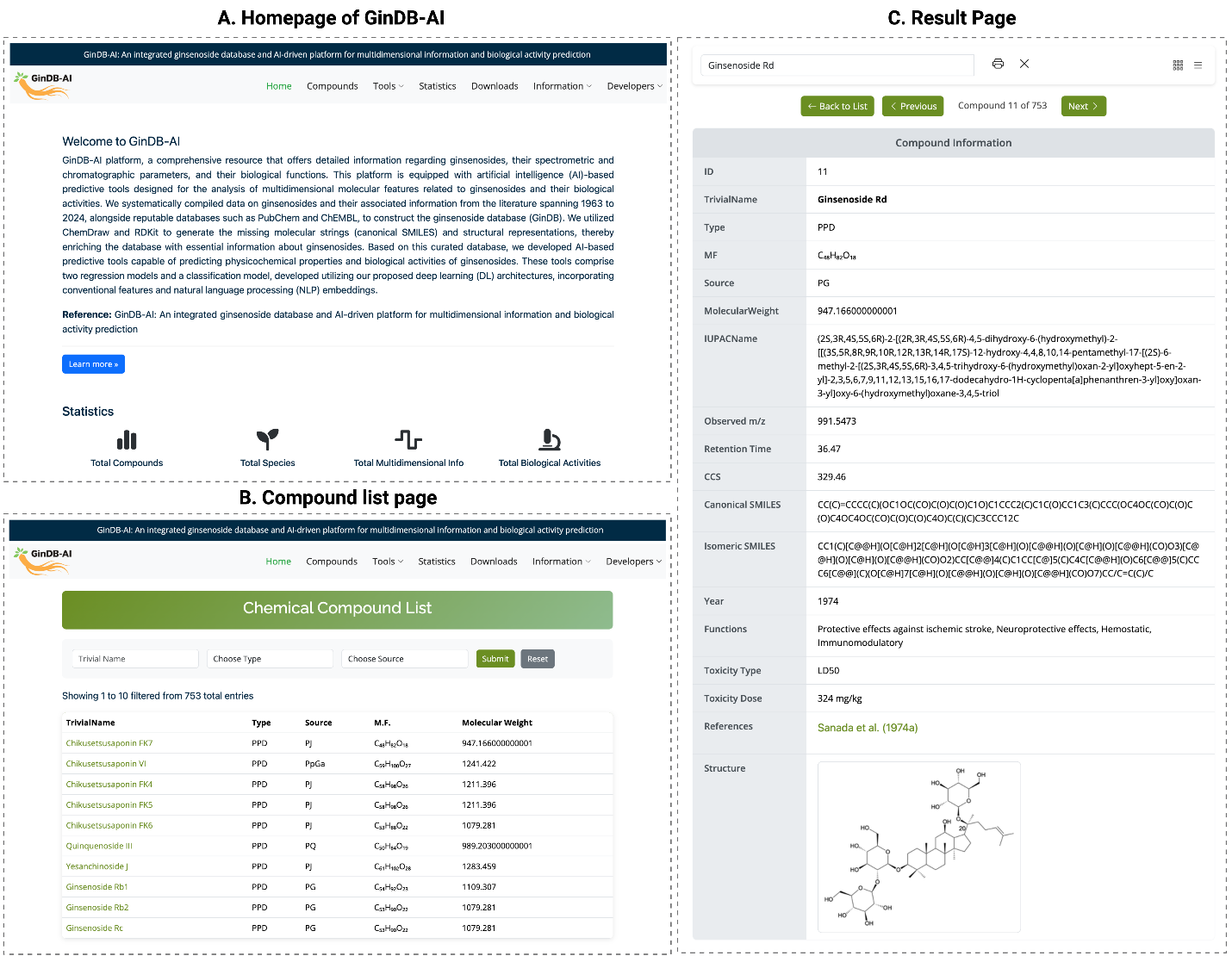


**Fig. S5.** The Homepage (A) shows the main entry page with navigation menu, database description, architecture of GinDB-AI, and real-time database statistics. The Compound list page (B) presents a searchable table displaying all ginsenosides that can be searched using compound name, chemical type, or botanical sources. The Result page (C) provides detailed information for each ginsenoside compound.

**References**

[1] Hall LH, Kier LB. Electrotopological State Indexes for Atom Types - a Novel Combination of Electronic, Topological, and Valence State Information. Journal of Chemical Information and Computer Sciences 1995;35: 1039-45.

[2] Gedeck P, Rohde B, Bartels C. QSAR− how good is it in practice? Comparison of descriptor sets on an unbiased cross section of corporate data sets. Journal of chemical information and modeling 2006;46: 1924-36.

[3] James CA. Daylight theory manual. <http://www>. daylight. com/dayhtml/doc/theory/theory. toc. html 2004.

[4] O'Boyle NM, Banck M, James CA, Morley C, Vandermeersch T, Hutchison GR. Open Babel: An open chemical toolbox. Journal of Cheminformatics 2011;3.

[5] Rogers D, Hahn M. Extended-Connectivity Fingerprints. Journal of Chemical Information and Modeling 2010;50: 742-54.

[6] Kim S, Chen J, Cheng TJ, Gindulyte A, He J, He SQ, Li QL, Shoemaker BA, Thiessen PA, Yu B, Zaslavsky L, Zhang J, Bolton EE. PubChem in 2021: new data content and improved web interfaces. Nucleic Acids Research 2021;49: D1388-D95.

[7] Landrum G, Tosco P, Kelley B, Rodriguez R, Cosgrove D, Vianello R, Sriniker, Gedeck P, Jones G, Kawashima E, NadineSchneider, Nealschneider D, Dalke A, Swain M, Cole B, tadhurst c, Turk S, Savelev A, Vaucher A, Wójcikowski M, Take I, Walker R, Scalfani VF, Faara H, Ujihara K, Probst D, Maeder N, Monat J, Lehtivarjo J, Godin G, 2025. rdkit/rdkit: 2025_03_3 (Q1 2025) Release (Version Release_2025_03_3). Zenodo. <https://doi.org/10.5281/zenodo.15605628>

<http://dx.doi.org/10.5281/zenodo.15605628>.

[8] Edwards C, Lai T, Ros K, Honke G, Cho K, Ji H. Translation between molecules and natural language. arXiv preprint arXiv:2204.11817 2022.

[9] Beltagy I, Lo K, Cohan A. SCIBERT: A Pretrained Language Model for Scientific Text. 2019 Conference on Empirical Methods in Natural Language Processing and the 9th International Joint Conference on Natural Language Processing (Emnlp-Ijcnlp 2019) 2019;3615-20.

[10] Chithrananda S, Grand G, Ramsundar B. ChemBERTa: large-scale self-supervised pretraining for molecular property prediction. arXiv preprint arXiv:2010.09885 2020.

[11] Yüksel A, Ulusoy E, Ünlü A, Dogan T. SELFormer: molecular representation learning via SELFIES language models. Machine Learning-Science and Technology 2023;4.

[12] Jaeger S, Fulle S, Turk S. Mol2vec: Unsupervised Machine Learning Approach with Chemical Intuition. Journal of Chemical Information and Modeling 2018;58: 27-35.

[13] Celma A, Bade R, Sancho JV, Hernandez F, Humphries M, Bijlsma L. Prediction of Retention Time and Collision Cross Section (CCSH+, CCSH–, and CCSNa+) of Emerging Contaminants Using Multiple Adaptive Regression Splines. J Chem Inf Model 2022;62: 5425-34.

[14] Wisanpitayakorn P, Sartyoungkul S, Kurilung A, Sirivatanauksorn Y, Visessanguan W, Sathirapongsasuti N, Khoomrung S. Accurate Prediction of Ion Mobility Collision Cross-Section Using Ion’s Polarizability and Molecular Mass with Limited Data. J Chem Inf Model 2024;64: 1533-42.

[15] de Cripan SM, Arora T, Olomí A, Canela N, Siuzdak G, Domingo-Almenara X. Predicting the Predicted: A Comparison of Machine Learning-Based Collision Cross-Section Prediction Models for Small Molecules. Analytical Chemistry 2024;96: 9088-96.

[16] Loshchilov I, Hutter F. Decoupled Weight Decay Regularization. arXiv [cs.LG] 2019.

[17] Khanzhina N, Lapenok A, Filchenkov A. Towards Robust Object Detection: Bayesian RetinaNet for Homoscedastic Aleatoric Uncertainty Modeling. arXiv [cs.CV] 2021.

[18] Lin T-Y, Goyal P, Girshick R, He K, Dollár P, 2017. Focal loss for dense object detection, Proceedings of the IEEE international conference on computer vision. pp. 2980-88.

[19] Yang WZ, Hu Y, Wu WY, Ye M, Guo DA. Saponins in the genus Panax L. (Araliaceae): a systematic review of their chemical diversity. Phytochemistry 2014;106: 7-24.

[20] Lou YY, Zheng X, Huang YP, Mu L, Zhang XG, Zhao ZW, Song Z, Zhang J, Yin ZQ, Pan K. New dammarane-type triterpenoid saponins from Gynostemma pentaphyllum and their Sirt1 agonist activity. Bioorg Chem 2021;116: 105357.

[21] Zhang M, Sun X, Ren R, Su L, Xu M, Zheng L, Li H. Six new dammarane-type triterpene saponins from the processed leaves of Panax notoginseng. Phytochemistry Letters 2022;49: 145-51.

[22] Li X, Liu J, Zuo TT, Hu Y, Li Z, Wang HD, Xu XY, Yang WZ, Guo DA. Advances and challenges in ginseng research from 2011 to 2020: the phytochemistry, quality control, metabolism, and biosynthesis. Nat Prod Rep 2022;39: 875-909.

[23] Lin Y, Hao B, Lu YC, Dong Y, Li Y, Zhang GH, Yang ZJ, Xiang GS, Liu GZ, Li XJ, Zhu Q, Yang QH, Li XZ, Yang SC. PanaxGDB: A Comprehensive Platform for Panax. Front Plant Sci 2022;13: 883818.

1. https://github.com/aspuru-guzik-group/selfies [↑](#footnote-ref-1)
